# A Feature-Space Theory of the Production Effect in Recognition

**DOI:** 10.1027/1618-3169/a000611

**Published:** 2024-07-30

**Authors:** Jeremy B. Caplan, Dominic Guitard

**Affiliations:** ^1^ Department of Psychology and Neuroscience and Mental Health Institute, University of Alberta, Edmonton, AB, Canada; ^2^ School of Psychology, Cardiff University, UK

**Keywords:** production effect, list-strength effect, recognition memory, selective attention, matched filter model

## Abstract

**Abstract:** Mathematical models explaining production effects assume that production leads to the encoding of additional features, such as phonological ones. This improves memory with a combination of encoding strength and feature distinctiveness, implementing aspects of propositional theories. However, it is not clear why production differs from other manipulations such as study time and spaced repetition, which are also thought to influence strength. Here we extend attentional subsetting theory and propose an explanation based on the dimensionality of feature spaces. Specifically, we suggest phonological features are drawn from a compact feature space. Deeper features are sparsely subselected from a larger subspace. Algebraic and numerical solutions shed light on several findings, including the dependency of production effects on how other list items are encoded (differing from other *strength* factors) and the production advantage even for homophones. This places production within a continuum of strength-like manipulations that differ in terms of the feature subspaces they operate upon and leads to novel predictions based on direct manipulations of feature-space properties.


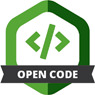


The prototypical production effect, where words read aloud are remembered better than words read silently (MacLeod et al., 2010), has been obtained with a healthy range of memory tests, including item recognition, free recall, cued recall, associative recognition, and serial recall (Bodner & MacLeod, 2016; Saint-Aubin et al., 2021). Theoretical accounts have mostly focused on two classes of mechanism (MacLeod et al., 2010): (1) *Strength Theory* assumes produced items are encoded more in memory and thus have a competitive advantage. (2) Inspired by Dodson and Schacter (2001), the *Distinctiveness Heuristic* holds that production during the study phase leads to participants sometimes remembering the act of production. They use this as the evidence they studied the word (“old”).

Mathematical modelers have begun to test how these principles might be concretely instantiated. To our knowledge, apart from the model we describe here, which was begun by Caplan (2023) and Caplan and Guitard (n.d.), there are only two published mathematical models of the production effect in recognition memory, an adaptation of MINERVA 2 (Hintzman, 1984) by Jamieson et al. (2016) and an adaptation of REM (Shiffrin & Steyvers, 1997) by Kelly et al. (2022, 2024) and just one for serial recall, an adaptation of the Feature Model (Nairne, 1990) by Saint-Aubin et al. (2021). These models all explain production advantages by assuming production results in more encoded features — namely features in a separate feature subspace that are encoded in produced conditions only. This improves memory by increasing effective strengths because the added features produce an effect similar to a scalar multiple of an encoded vector, a conventional way of modelling strength. But it also increases distinctiveness of encoded items because the additional features will differ in their values across items. Thus, mathematical models speak to both Strength Theory and the Distinctiveness Heuristic via a single mechanism and have been able to explain a large range of empirical findings related to the production effect.

Our model inherits these design principles from those models, which we elaborate as we introduce the mathematical formulation of our vector representation and attentionally subsetted adaptation (Caplan, 2023; Caplan & Guitard, n.d.; Caplan et al., 2022) of the matched-filter model (Anderson, 1970). More detailed derivations can be found in Caplan (2023) and Caplan and Guitard (n.d.). For exposition purposes, unless otherwise noted, we assume production is via vocalization, so phonological features are modulated by production. The model can easily be generalized to other forms of production by swapping out the functions of the feature subspaces, for example, production by typing would presumably influence orthographic features and not so much phonological features. This makes our account appropriate across the impressive continuum of “production” (MacLeod & Bodner, 2017) that has found similar findings with typing, writing, and even whispering (albeit with smaller effects).

As we fully unpack in the Discussion, Strength Theory was challenged because production did not seem to function like other manipulations of strength, particularly in old/new recognition, where participants judge whether each probe word was on the list (target) or not (lure). In recognition, standard ways of “strengthening” items, such as spaced repetitions and lengthening study duration, show a so-called null list-strength effect. A list-strength effect describes a large effect of strength on memory success when they are mixed within a single list but which becomes quite small when lists are composed only of one strength. Because strength leads to a relative competitive advantage, competing against other strong items cancels out most of the benefit of increased strength of a given item, so pure nonproduced lists are, overall, nearly as accurate as pure-produced lists (Caplan, 2023 showed this in model version 6, as did Jamieson et al., 2016). Thus, the *strength of the list* (strengths of other items within the list) matters to recognition of a given item. This result is found for production (Bodner et al., 2016; Hopkins & Edwards, 1972; MacLeod et al., 2010) but not (or minimally) for traditional manipulations of strength (Ratcliff et al., 1990). If repetition, stimulus duration and production all influence memory in the same way, one would expect these manipulations all to produce similar effects. Our main proposal here is that we may be able to explain why production differs from other strength manipulations not by assuming different processes, but by closely examining the characteristics of the feature-spaces likely influenced by these experimental factors. The characteristics of the affected feature subspaces may explain the ways in which production differs from stimulus duration and repetition. The consequences of production in a given experiment will depend specifically on how many features are stored, the size and properties of the production feature-space, and how this relates to other relevant feature subspaces.

We focus on recognition because of the need to explain the diversity of list-strength effects, which is less controversial for other tasks (Ratcliff et al., 1990). Also, the simplicity of recognition distills memory down to the pattern of similarity (and confusability) between items in memory. Developing our ideas with recognition lets us more easily trace the consequences of our assumptions through the mathematical derivations. However, the insights will carry through to other memory tasks because they depend on these same principles of similarity of vector representations of items.

Next we describe our model, how it derives from previous model and what it adds. We then apply the model to several empirical phenomena that have otherwise not been addressed by models. We show how attentional subsetting theory can explain large list-strength effects with production in contrast to other strength manipulations, whether there should be a speed–accuracy tradeoff with production, and whether production effects can be influenced by semantic aspects of words without needing to assume production acts directly on semantic features.

## Conceptual Walk-Through and Summary of the Model

**Figure 1:**
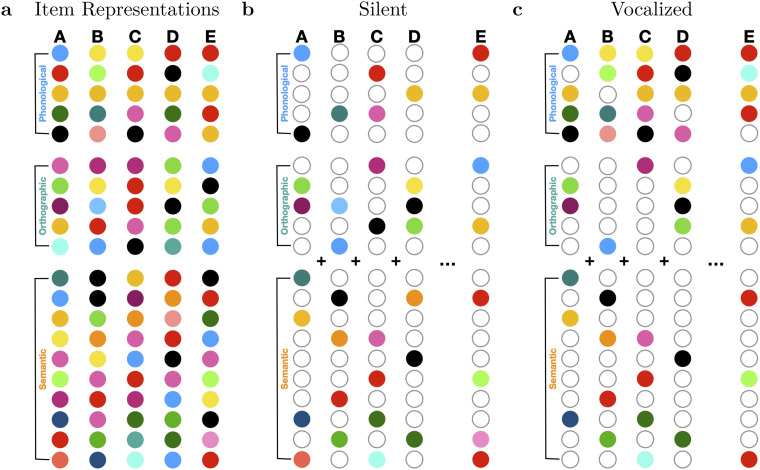
Schematic depiction of item representations and how they differ across production conditions. (a) The full vector representation of five items (i.e., “lexicon” or “knowledge”). Each circle represents a feature and the color of the circle denotes its numerical value (arbitrary scale). We depict two (but there could be more) subsets of “shallow” features, phonological and orthographic. We depict one larger-dimensional feature subspace to stand in for deeper features such as those related to semantics/meaning, imagery, etc. (b) Silent condition (words are not “produced”). Grey unfilled circles denote features that are not attended (and thus not encoded). We assume that the shallow features are dense, not sparsely subsetted, whereas the deep features are sparsely subsetted. In the silent condition, we assume that typically, some deep features, some orthographic and some phonological features are attended (those that are colored in) and thus encoded in the memory. The example list here consists of items A through D, where the memory is their sum, A + B + C + D. E is an example of a lure probe. (c) Vocalized condition (words are “produced,” by reading aloud, in this example). Because production is an additional process the participant is instructed to do, we assume that this results in more features overall attended and encoded (more colored circles in panel c than in panel b). Most of the extra features are phonological. We also allow for a tradeoff (but we do not implement it in this manuscript); production might partly displace attention and encoding of both orthographic and deeper features, so for some items, fewer orthographic and/or fewer semantic features are colored in. Note that for pure lists (all silent or all vocalized), we assume that the probe item is attended in the same way as if it had been a studied item; the participants’ meta-knowledge leads them to seek more phonological evidence following vocalized lists and less phonological evidence following silent lists. For mixed lists, there mask might be a one or the other or a union or mixture of the two.

In plain terms, we consider each item to consist of a set of features (Figure 1). As reasoned by Caplan (2023), the number of features representing full knowledge of a word must be quite large (tens of thousands to avoid linear dependence). It seems implausible that in an episodic memory task, we process and encode all such features (and indeed, typical vector models of memory function within a low-dimensional working space, with tens or hundreds of features). Rather, we assume that when studying an item, one attends to a small subset of all known features of an item.^1^ Only attended features can be encoded. Those features will be particular to the item and differ across items. But in addition, task conditions can bring attention toward or away from particular kinds of features, which we refer to as feature subspaces. When reading aloud, more phonological features are attended than when reading silently. These subsetted feature vectors get added up in a memory structure.

Then recognition is done by comparing the probe item to memory. The more features of the probe match features stored in memory, the greater the matching strength will be. This already explains some of the production effect, common to the previous mathematical models. Produced items will tend to have more features stored in memory and thus available to match, like strength, but the additional features will also be somewhat item-specific, adding distinctiveness. But we also assume people process the probes similarly to how they studied the list. The probe thus also consists of a subset of features. In a pure list (all silent or all aloud), the subsets will be consistent with the subsets during the study phase, but when tested on a mixed list, the participant needs to decide whether to process a probe item as though it were aloud or silent or some combination of both.

Finally, the added features offered by production are thought to be accessed early in processing an item. Production thus enhances memory by acting on early-accessed features. In contrast, other manipulations of strength may enhance memory through additional encoding of features that take longer to access, such as semantic or imagery-related features. This may explain why production increases accuracy without a cost to response time whereas other strength manipulation produces a speed-accuracy tradeoff.

## Assumption 1: Features of Different Types Should Be Distinguished

All three prior models explicitly separate item features into subclasses, and in particular, those related to production and those unrelated to production (Figure 1a). Let each word be an *n*-dimensional vector, **f**_*i*_, where boldface denotes (column) vectors and the subscript indexes unique word. Each feature of word *i* is indexed by *k* in parentheses, *f*_*i*_(*k*). One can think of very concrete properties of words, such as TURTLE—feature “animacy” = true, feature “color” = green, feature “appearance” = cute, etc. In general, features are not specified; they are treated as mathematical entities, and thus, in our derivations and simulations, we generally assume features are independent, identically distributed (i.i.d.), in other words, each drawn at random from a normal distribution^2^ with *M* = 0 and variance = 1/*n*. We implement Assumption 1 by partitioning the *n* features into a number of feature subspaces. Let *n* denote the full set of features that are considered in the model (i.e., the maximum set of features that might be relevant for the task) and *n* with a subscript denote the number of features within a given subspace. We define:

$$\begin{array}{l} n_{\pi }=\text{Number of phonological features}\\ n_{\omega }=\text{Number of orthographic features}\\ n_{\sigma }=\text{Number of semantic features}, \end{array}$$

depicted in Figure 1a. To keep things organized, we fix *n* = *n*_*π*_ + *n*_*ω*_ + *n*_*σ*_ so that no features are unassigned to a subspace, and we number the features consecutively; phonological features: *k* = 1…*n*_*π*_, orthographic features: *k* = (*n*_*π*_ + 1)…(*n*_*π*_ + *n*_*ω*_); semantic features: *k* = (*n*_*π*_ + *n*_*ω*_ + 1)…*n*. This idea is not new; models have long drawn distinctions specifically among phonological, orthographic, and semantic features (e.g., Burgess & Hitch, 1999; Seidenberg & McClelland, 1989).^3^ We build from the very simple matched filter model introduced by Anderson (1970), where the memory is a vector, **m** that is simply a sum of the *L* (list length) vectors representing list-items,

$$m=\overset{L}{\underset{i=1}{\sum }}f_{i}.$$

Normally a scalar value would multiply each term to add variability in encoding strength. We omit these so that the derivations remain clear and easy to follow. Recognition is done by computing the dot product between a probe-item vector, **f**_*x*_ and the memory vector to obtain a matching strength, for the probe item, *s*_*x*_,

$$s_{x}=f_{x}\cdot m$$

The model compares this strength to a threshold, *θ*, also call a criterion, and responds

$$\left\{ \begin{array}{cc} “\text{Old}” & s_{x}>\theta \\ “\text{New}” & s_{x}\leq \theta \end{array}\right.$$

One can compute the mean matching strength of lures and targets (Anderson, 1970),

$$\mu_{\text{lure}}=E\left[s_{\text{lure}}\right]=0$$

$$\mu_{\text{target}}=E\left[s_{\text{target}}\right]=1,$$

where E[ ] denotes the expectation (*M*) and variances are denoted var[ ]. Strength variances can be calculated in two steps. First we write *V*_*xx*_, the variance for the case of *i* = *x*, the encoded term that matches the probe item. There will be only one of those for target probes and none for lure probes. We also write *V*_*xy*_, *x* ≠ *y*, the cross-term between the probe item and all terms associated with encoding of nonmatching items. The lure has only such terms, *L* of them. The target has *L* − 1 of them.

$$V_{xy}=\text{var}\left[s_{\text{lure}}\right]=1/n$$

$$V_{xx}=\text{var}\left[s_{\text{target}}\right]=2/n,$$

as derived elsewhere (Caplan, 2023; Weber, 1988). Adding up the variance contributed by each encoded item’s term,

$$\sigma_{\text{lure}}^{2}=LV_{xy}=L/n$$

$$\sigma_{\text{target}}^{2}=V_{xx}+\left(L-1\right)V_{xy}=2/n+\left(L-1\right)/n=\left(L+1\right)/n,$$

and the feature subspaces are handled easily because the means and variances summate, so *μ*_target_ = (*n*_*π*_/*n* + *n*_*ω*_/*n* + *n*_*σ*_/*n*) and *V*_*xy*_ = (*n*_*π*_/*n* + *n*_*ω*_/*n* + *n*_*σ*_/*n*) (1/*n*), etc.

## Assumption 2: Production Influences Encoding of Production Features in Particular

The previous models also assumed that production increases encoding of production-related features—phonological features in the case of vocal production (depicted in Figure 1b, c) or orthographic features in the case of typed production (or motor codes, which we consider in the Discussion). For this to make sense, it comes with the assumption that not all *known* features of each item are stored, which departs from the original matched-filter model but was introduced by Murdock (1982). An unencoded feature is multiplied by zero, which in attentional subsetting theory, we think of as an attentional mask (Caplan, 2023; Caplan & Guitard, n.d.; Caplan et al., 2022).

Whereas *n*_*π*_, *n*_*ω*_ and *n*_*σ*_ denote the full feature subspaces, respectively, *ν*_*π*,*c*_, *ν*_*ω*,*c*_, and *ν*_*σ*,*c*_ will denote the number of features attended within each subspace: the corresponding *attentional subset.* We also index the *ν* variables by *c*, standing in for the task condition. So in this notation, we denote the idea that production condition results in more phonological features attended (and encoded) than the nonproduction condition like this: *ν*_*π*,aloud_ > *ν*_*π*,silent_, illustrated in Figure 1, comparing panel c to panel b.

Considering, for a moment, the full vector, let F_*i*,*c*_ denote the set of attended features of item *i* under conditions *c*. The attentional mask, **w**_*i*,*c*_ is an *n*-dimensional vector with at each dimension included in F_*i*,*c*_ and 0 elsewhere:

$$w_{i,c}\left(k\right)=\left\{ \begin{array}{cc} 1 & k\;\in \;F_{i,c}\\ 0 & k\;\notin \;F_{i,c} \end{array}\right.$$

and this mask vector then simply multiplies elementwise (denoted ⊗) the corresponding item vector before encoding, so Equation 1 becomes

$$m=\overset{L}{\underset{i=1}{\sum }}w_{i,c}⊗f_{i}.$$

The same elementwise multiplication is applied at test, either with the same **w**_*i*,*c*_ or in some circumstances with a different mask. Because the means and variances for each feature subspace simply add, we can partition the model by subspace (*π*, *ω* and *σ*) and then add the results at the end.

A scalar multiple of a vector increases the vector’s *strength* by increasing its length. But if unencoded features multiply by zero, then if more features are encoded, that also increases its length. If a production condition increases the number of features stored, one effect is thus to increase the length and thus the effective strength of the encoded memory (Caplan, 2023). At the same time, encoding more features will tend to increase the distinctiveness of one studied item from another and in the case of differentiation models (Criss, 2006; Shiffrin & Steyvers, 1997), even of the studied items from the lures. The additional features thus capture both strength and distinctiveness.

Where previous modelers have already found value in segregating out production-related features, we add a few additional concrete assumptions about the nature of production-feature spaces versus feature spaces comprised of different types of information. The other models of production effects are all local-trace models. Here we formulate our ideas and demonstrate them in an attentionally masked matched-filter model (a simple vector sum of studied items—after applying an item-specific attentional mask). We do this not to argue against other models (nor local-trace or differentiation assumptions) but because the matched-filter model is mathematically extremely simple—so simple that its limitations are easy to identify and well known. It distills recognition memory down to the effective similarity relationships between items. This makes it easy to understand how it works through both analytic derivations and simulations. And because any model that starts with representations of items as sets of features is really a vector model, most of the insights gained will propagate when attentional subsetting is implemented in those more complete, fleshed-out models. The framework we work within, attentional subsetting theory, aside from the production effect, has been shown to explain why near-null list-strength effects are so common in recognition, why small positive list-strength effects are expected, and how even inverted list-strength effects (larger effect of strength in pure than in mixed lists) can arise (Caplan, 2023; Caplan & Guitard, n.d.). Caplan and Guitard (n.d.) also showed how a response threshold (criterion) could be tuned based on immediate processing of the current probe, with the potential to produce symmetric strength-based mirror effects (but also asymmetric ones in some conditions), without requiring local traces, differentiation, or unrealistic knowledge of expected strength distributions. Those were continuum accounts of list-strength effects and strength-based mirror effects. Likewise, our account of production effects in recognition will be a continuum account, with guideposts as to which factors could relatively enhance or reduce production-based advantages.

## Assumption 3: Sparseness of the Attentional Subset Matters

New to models of the production effect, attentional subsetting theory assumes production features have different properties than other features such as semantic, imagery-based, etc (Caplan, 2023; Caplan & Guitard, n.d.). Here we investigate the idea that a few formal assumptions about the characteristics of produced features, and how they differ from other features, can explain why a production effect occurs and why it produces a list-strength effect and a mirror effect, but also why certain factors attenuate it, such as long study time. Jamieson et al. (2016) viewed production effects as belonging to a family of phenomena including the generation effect (when participants are indirectly cued to think of the item) and the enactment effect (acting out items) and we align with this. In line with Jamieson and colleagues, we do not view production effects as categorically different or special cases compared to other phenomena, but as particular conditions. The peculiarities of the production effect are not due to the peculiarity of production but to the particularities of the feature-subspace that production emphasizes.

In our formulation, we assume the attentional subsets within the different feature spaces, which we denote *ν*_*π*,*c*_, *ν*_*ω*,*c*_ and *ν*_*σ*,*c*_, respectively, may not be identical in number but are roughly of the same order of magnitude. That is, people tend to attend to a handful of phonological features, a handful of orthographic features, and a handful of semantic features of a given item. To explain why list-strength effects are large for the production effect but small for other manipulations of strength (Caplan, 2023), we assume that *n*_*σ*_ ≫ *n*_*ω*_ ≃ *n*_*π*_. This means that a subset of features attended on a given item, *i*, in condition *c*, will be a sparse subset for semantic features, which we write F_*σ*,*c*,*i*_. The *σ* features are masked by multiplying every feature by zero if it is not contained in F_*σ*,*c*,*i*_. Because *ν*_*σ*,*c*,*i*_ ≪ *n*_*σ*_, the chance of there being semantic features common to two items, *i* and *j*, is extremely small and nearly zero. Sparseness causes the *V*_*xy*_ terms to be mostly zeroes as well. *V*_*xx*_ remains unaffected by sparseness; it is only influenced by the number of attended features, *ν*_*σ*,*c*,*i*_. Essentially, sparseness reduces noise due to “cross-talk” from other studied items, and leads to very minimal influence of other list items on recognition of a probe.

What makes sparse subsetting work is that we also assume that the subset of features attended during study will tend to be quite similar to those attended when the same word later appears as a probe (a target), itself. The idea is that if seeing the word HUMMINGBIRD causes you to think of the hovering, fast wings, iridescent coloring and sharp beak when you first see the word, then when you later encounter HUMMINGBIRD again, you will very likely attend to those same features. It is a principle of (approximate) tautology; features that come to mind readily at one time are likely to come to mind readily at another time. This tendency needs to be explained in a full model of semantic memory or knowledge, but here we only presume its existence. Some support for the consistency of attended features across participants on the one hand, and their modulation by task demands on the other, can be found in explicit feature-listing experiments (Wu & Barsalou, 2009) and similarity and prototypicality judgements (Medin & Shoben, 1988).

For *shallow* features, including phonological and orthographic features, we assume that the feature space is far smaller and as a consequence, subsetting a handful of features will not be sparse. This means there is a large amount of chance-overlap between the attended feature subsets of one item and another; F_*σ*,*c*,*i*_ ∩ F_*σ*,*c*,*j*_ is non-negligible when *j* ≠ *i*. Each of those common features increases *V*_*xy*_, adding to noise contributed by the cross-terms (other studied items).

Hit and false-alarm rates. Next, by adding the criterion heuristic suggested by Caplan and Guitard (n.d.), we can apply a threshold, *θ*_*i*,*c*_ (recall that *c* stands for an experimental condition) and solve for the hit rate and false-alarm rate separately. The theory already assumes participants process the probe very much as they would have if the probe item had been presented during the study phase, including application of an attentional mask, **w**_*i*,*c*_, before computing similarity by dotting the masked vector with the memory vector, **m**. As Caplan and Guitard (n.d.) reasoned, it is plausible that the participant has access to the (approximate) number of features they attended on the probe item, itself, *ν*_*i*,*c*_, given that this feature-extraction is happening in real-time. This could be as overt as a rough count of the number of features that come to mind in response to the current probe, or it might be more of a vague feeling about how much matching there might be (this could be tested in future experiments), but seems to demand less of the participant than expecting participants to have accurate access to characteristics of the memory, itself, or cumulative knowledge about what happened on other test trials. Given this, the participant can then straight-forwardly compute an optimal value halfway between the expected mean strength for targets (*μ*_target_) and lures (*μ*_lure_):

$$\theta_{i,c}=\left(1/2\right)\nu_{i,c}/n.$$

For pure silent and aloud lists, these will be:

$$\theta_{i,\text{pure silent}}=\left(1/2\right)(\nu_{\omega }+\nu_{\sigma })/n$$

$$\theta_{i,\text{pure aloud}}=\left(1/2\right)(\nu_{\omega }+\nu_{\sigma }+\nu_{\pi })/n.$$

For mixed lists, the threshold might be based on the smaller or the larger of the two conditions or an average of the two. For duration, we have assumed the threshold is based on the larger of the two (Caplan & Guitard, n.d.), but as we shall see in the first fit of the model to production-effect data, the model fit substantially better when the average threshold was used. Since we have already solved for the variances that target and lure items are subject to, we can then compute the hit rate and false-alarm rate by integrating the respective normal distribution from the corresponding threshold upward to infinity (Caplan & Guitard, n.d.).

Outline. Having introduced the model and the core assumptions, next we solve for the hit rate (proportion of target items responded “old”) and false-alarm rate (proportion of lure items responded “old”) as well as *d*^′^ (the measure of sensitivity derived from signal detection, *z*-transformed hit rate minus *z*-transformed false-alarm rate). We see how the assumptions lead to a list-strength effect and a strength-based mirror effect. We compare this to a model-manipulation of stimulus-duration. This incorporates the assumption that shallow features are processed and attended earlier than deeper features (e.g., Caplan, 2023; Caplan & Guitard, n.d.; Gardiner et al., 1999; Mulligan & Hirshman, 1995). We then look at the effects of extending stimulus duration on a production manipulation, itself. We briefly address data from Fawcett et al. (2022) that was used to argue that production influences encoding of more than production-related features. Then we wrap up with a re-evaluation of Strength Theory and the Distinctiveness Heuristic in light of our account.

## Attentional Subsetting Model of the Production Effect: Production Increases the Number of Encoded Shallow Stimulus Features

Caplan and Guitard (n.d.) modelled an experimental manipulation of stimulus duration, by assuming that words presented for longer (e.g., 2 s) resulted in more deep features attended and encoded than words presented for shorter durations (e.g., 1 s). They assumed the number of shallow features was equivalent between long and short conditions. To model the production effect, we turn this around. We assume no difference in the number of deep (semantic) features encoded and no difference in the number of orthographic features. In the core model we explore here, the only thing that will differ during the study phase is that words read aloud will have more phonological features encoded than words read silently; as we already wrote above, *ν*_*π*,aloud_ > *ν*_*π*,silent_.

### Fit to the List-Composition Experiment of Bodner et al. (2014)

We start by examining what happens in list-composition experiments. In the Discussion, we fully unravel the recent history of so-called “list-strength effects” and the role played by research on the production effect. Briefly, in a list-composition experiment, two item-conditions are either segregated to pure lists or mixed (usually half of each condition) in one list. If the experimental manipulation affects the encoded strength of an item (an assumption that is often presumed but not directly tested), this is called a list-strength manipulation. If there is any competition amongst studied list items during recognition tests, then the advantage of strong over weak items is expected to be greater in mixed lists than in pure lists. Ratcliff et al. (1990) were surprised when the expected list-strength effect was not found. However, Caplan (2023) argued that the list-strength effect was not absent, but rather, quite small. Sparse subsetting could explain the small magnitude of list-strength effects, as well as why they might sometimes invert (greater effect of strength in pure than in mixed lists, elaborated by Caplan & Guitard, n.d.) and alluded to the production effect as being a possible counter-example, where positive and quite large-magnitude list-strength effects are in fact observed (Bodner et al., 2014, 2016; Hopkins & Edwards, 1972; MacLeod et al., 2010). Here we test that suggestion in an implementation of the production effect in the attentional subsetting model.

First we fit data from a list-composition production effect study reported by Bodner et al. (2014) who had participants study lists of 50 words, presented for 2.5 s each (including a 0.5 s blank inter-stimulus interval). Each word was either read aloud or read silently. Their experiment had more conditions than we were interested in; we fit the mean hit rate and mean false alarm rate for pure-aloud lists, pure-silent lists and mixed lists, separated by whether the word was aloud or silent. Naturally, false-alarms for mixed lists were not broken down by aloud/silent. This produced seven independent data points that we fit the model to.

The model potentially has a very large number of parameters one could treat as free parameters in a parameter optimization. And yet, as stated earlier, it is not meant to be a complete model of recognition. The goal here was not to fit the data perfectly, but to check whether the model could approach real empirically observed values and capture the qualitative features seen in the data. Thus, we somewhat arbitrarily (and with some continuity with previous explorations of the model) fixed the number of semantic features, *n*_*σ*_ = 512, the number of shallow features, *n*_s_ = 128 such that half of those were devoted to orthographic features and half to phonological, *n*_*ω*_ = *n*_*π*_ = 64. The total vector length was thus *n* = 640. We conducted a direct search (comparing all combinations of integer parameter values) of a three free-parameter space, varying the number of subsetted features as follows. For semantic features, *ν*_*σ*_ varied from 1 to 32 features and this applied to both aloud and silent words. For orthographic features, *ν*_*ω*_ varied from 1 to 64 (*n*_*ω*_) and applied to both aloud and silent words. *ν*_*π*_ also varied from 1 to 64 (*n*_*π*_) but we assumed phonological features were only stored while studying a word aloud. This is not to claim that the silent condition results in zero encoding of phonological features, it is just a simplification to keep the number of free parameters low. We also assumed that in the pure-silent condition, participants would disregard the phonological features. This reduces the criterion used in pure-silent lists, which offsets some of the reduction in hit rate due to fewer features being encoded. The complete three-dimensional parameter space was solved for hit and false alarm rates, then root-mean-squared deviation was computed relative to the data and the maximum log-likelihood parameter-set was identified. As proposed by Caplan and Guitard (n.d.), response criterion, *θ*, was determined by dividing the number of attended features (*ν*_*σ*_ + *ν*_*ω*_ + *ν*_*π*_ for a given condition) in half, multiplying 1/n for scale (approximate normalization).

**Figure 2:**
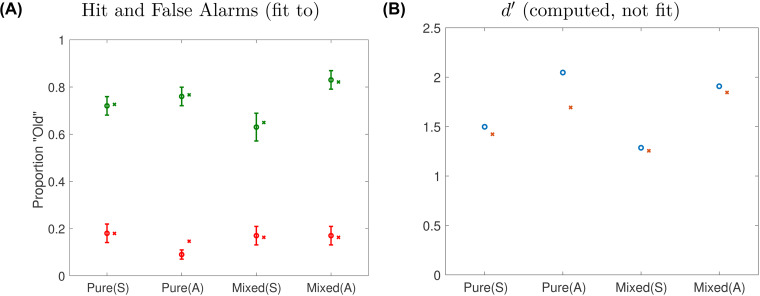
(**A**) Fit of the model (denoted with x) to data (denoted with o, with error bars denoting the 95% confidence interval based on standard error of the mean) to the hit rate (green) and false alarm rate (red) in each of the four experimental conditions. (B) *d*^′^ computed from the model (denoted with x) and the data (denoted with o). Note that the *d*^′^ values were not fit to. Data *d*^′^ were computed from the authors’ reported hit and false alarm rates, which the model was fit to, rather than the authors’ reported *d*^′^ values, which showed the same qualitative pattern but were larger. For model parameter values, see main text.

The best-fitting parameter set, with log-likelihood = 52.69, had *ν*_*σ*_ = 2, *ν*_*ω*_ = 3 and *ν*_*π*,aloud_ = 3. This was assuming that for mixed lists, *θ* was the average of that used for the pure-silent condition and that used for the pure-aloud condition. When the search was re-run using the criterion for pure-silent lists, log-likelihood was considerably lower (46.44), indicating a quantitatively worse fit. The fit was also worse (log-likelihood = 47.36) if we used the criterion for pure-aloud lists. Figure 2a plots the model and data hit rate and false alarm rate in each condition. The hit rates are fit well, and even within the confidence intervals, and importantly, they reproduce the list-strength effect, where the advantage due to production is greater within mixed than within pure lists. The false alarms are fit well, although the rate for pure-aloud is overestimated. Still, if one looks closely, the rank-order of the three false-alarm rates is reproduced by the model. Without any refitting, when we plot *d*^′^ computed from the hit and false-alarm rates, the qualitative pattern is reproduced by the model (Figure 2b), especially the list-strength effect, where the advantage due to production is greater in mixed lists than in pure lists.

Curiously, the model produced good fits using very few subsetted features within each subspace. It is possible that this is a reasonable estimate of the effective number of features that participants actually attend under these conditions (e.g., total study time available was 3 s/word). Recall, however, that this model includes no variability and no noise; in a more complete model, more features would presumably be needed to overcome noise to match realistic performance levels. Also, consider that if three were the mean number of features attended, that implies that some words have fewer, even sometimes no features attended within a given feature subspace, and occasional item may have a lot of features attended, averaging out to 3, which strikes us as plausible considering the swift presentation rate. In any case, these fits can be seen as proof of principle that the idea that only a handful of features are encoded for each item is sufficient both to produce performance (*d*^′^, hit rate and false-alarm rate) in the observed ranges but also the capture some key qualitative features of the data.

### Sensitivity to Parameters and Experimental Factors

With this best-fit to the Bodner et al. (2014) data as a reference model, we next vary parameters to explore the sensitivity of hit rate, false-alarm rate and *d*^′^ to hypothetical experimental manipulations.

The criterion used for mixed lists was again the average of that for pure-silent and that for pure-aloud lists. The feature spaces were fixed at *n*_*ω*_ = *n*_*π*_ = 64 and *n*_*σ*_ = 512.

**Figure 3:**
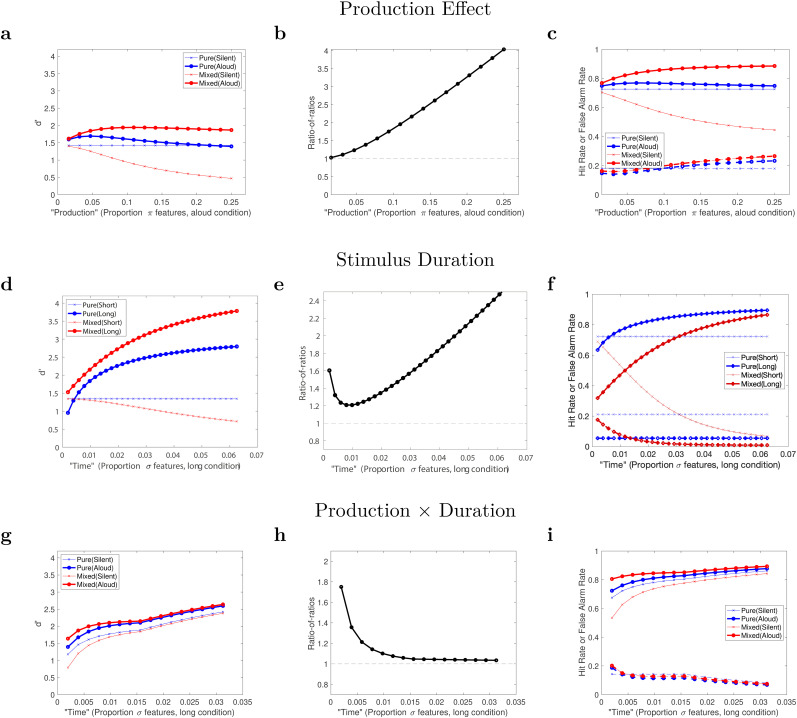
Sensitivity of the model to amount of production, stimulus duration and the interaction of the two. Top row: the effect of increasingly more production (more phonological features attended and encoded), while holding constant the silent condition. *L* = 50, *ν*_ω_ = 3, *ν*_σ_ = 2 and *ν*_π,aloud_ ranges from 1 to 16 (out of 64), plotted in proportion of *n*_π_ (64) in the *x* axis. Note that for encoding of the silent items and recognition of pure-silent lists, *ν*_π,silent_ = 0 and the *x* axis does not apply. Middle row: the effect of increasing stimulus duration of the longer-duration condition (more deep, or semantic features encoded), while holding constant the short-duration condition. For all words, shallow features (combining orthographic and phonological) had *ν*_s_ = 6 out of *n*_s_ = 128 features stored, and for the long duration only, an additional n_σ_ features were stored, ranging (on the *x* axis) from 1 to 32. Bottom row: the effect of increasing stimulus duration on a putative production-effect manipulation, comparing silent to aloud conditions. *ν*_π,aloud_ = 3 and *ν*_π,silent_ = 0. *ν*_σ_ was varied from 1 to 16 and *ν*_ω_ = *ν*_σ_ but capped at 8. The left column of panels plots *d*^′^ and shows list-strength effects nearly throughout. These list-strength effects are reflected in positive ratio-of-ratios, plotted in the middle column. The right column of panels separates the model’s calculations of hit rate and false alarm rate, showing mirror effects whose magnitude and (a)symmetry are parameter-dependent.

**Figure 4:**
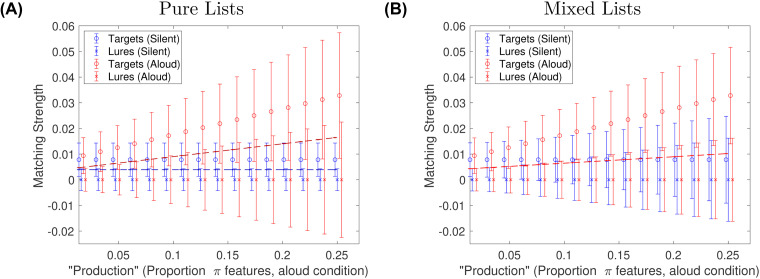
Visualization of distributions of matching strength, which is the match of a probe (attentionally subsetted) to memory. It is from these distributions that performance is computed (hit rate, false-alarm rate and *d*^′^). Corresponding to the production effect sensitivity check plotted in Figure 3a–c, these graphs plot mean matching strength and standard deviation (i.e., √variance, depicted in the error bars), for targets and lures, for pure-list (A) and mixed-list (B) conditions, respectively. θ for silent and aloud probes are plotted in dashed lines in the same color as the matching strengths, themselves, but note that for mixed lists, only one threshold line is visible because the threshold does not vary between conditions. Also note that the *x* values have been shifted slightly to avoid error bars obstructing each other.

Results of the simulation of the list-composition manipulation that is done in list-strength effect studies are plotted in Figure 3a–c for a hypothetical manipulation of production (for a visualization of the means, variances and thresholds that were used to compute these, see Figure 4). As more phonological features are attended in the aloud condition, moving from left to right in panel a, the bigger the production effect (difference between the red plots) becomes within mixed lists. Recall that we are assuming participants match probes on phonological features when tested on mixed lists. For this reason, the presence of phonological features in mixed lists, combined with the fact that the model cannot disregard those, puts silent items at an increasingly bigger disadvantage. Aloud items benefit more from being in mixed lists as we move from left to right, but *d*^′^ values saturate when just a few percent of the phonological features are attended per word (this number may seem low, but recall that 50 words are encoded). For pure lists, the picture is different. For very few attended phonological features, more phonological features leads to more of a production advantage. Once a few percent of the available phonological features are encoded for each item, performance on aloud words starts to decrease. This can be understood as an accumulation of cross-talk due to massive similarity of the phonological features with other items. In pure-aloud lists, more phonological features (twice as many, because there are twice as many aloud items) are stored than in pure-silent or in mixed lists, allowing this disadvantage due to cross-talk to emerge. The ratio-of-ratios (panel b) was defined by Ratcliff et al. (1990) as the ratio of the “strength” (here, production) effect in mixed lists divided by that in pure lists, thus:

$$\mathrm{RoR}=\frac{d^{\prime }\left(\text{Mixed Strong}\right)/d^{\prime }\left(\text{Mixed Weak}\right)}{d^{\prime }\left(\text{Pure Strong}\right)/d^{\prime }\left(\text{Pure Weak}\right)}$$

so that a null list-strength effect would have *RoR* = 1, and the expected (positive) list-strength effect would have *RoR* > 1. Across the explored parameter range here, *RoR* > 1 throughout, but when the production manipulation is weaker, it converges toward an approximate null effect, *RoR* ≃ 1 (left portion of the plot).

For pure lists, we can also check for a mirror effect; that is, do hits increase and false alarms decrease together as more phonological features are attended? Figure 3c (blue plot lines) shows that for low amounts of production (left portion of the graph), hits are greater and false alarms are reduced by a comparable amount, an approximately symmetric mirror effect. At higher levels of production (rightward on the graph), false alarms cross over so that the aloud condition produces more false alarms than the silent condition, despite the aloud condition producing greater *d*^′^ overall (see panel a). With even more production features stored, the hit rate and false alarm rate both move toward the center, producing a kind of reverse mirror effect which, with these parameter values, also neutralizes the production effect altogether (see the blue plots in the right portion of panel a).

This simulation illustrates that although this account of the production effect is quite simple (only the number of encoded phonological features differs between silent and aloud processing), slight variations in parameter values can change features of the results that would be deemed to be theoretically informative qualitative features. Specifically, we have shown that the list-strength effect can be positive or near-null and there can be an approximately symmetric mirror effect, or production can primarily influence the hit rate or even produce a reverse mirror effect. One intuitive lesson we can draw from this is that due to the assumed compactness of the phonological feature-space, the advantage due to production can often be offset or cancelled out, or possibly even reversed, due to increases in false alarms that occur because the phonological features are maxed out and produce a large amount of cross-talk interference. This sometimes self-sabotaging characteristic of production in pure lists may also explain why pure-list production effects have often not been confirmed and why meta-analysis was needed to establish their at least occasional (very) robustness (Bodner et al., 2014; Fawcett, 2013).

For comparison, Figure 3d–f plots a simulation of a manipulation of stimulus duration. This was adapted from the simulation in Caplan and Guitard (n.d.) but with parameters closer to our fit of the Bodner et al. (2014) data. In this simplified simulation, it was assumed that longer duration leads simply to more deep (such as semantic) features encoded. Thus, *ν*_*σ*_ was varied from 1 to 32 (out of *n*_*σ*_ = 512 total features) for the long duration condition and the shallow condition had zero semantic features stored. Both long and short duration items were assumed to have *ν*_s_ = 6 ( = *ν*_*ω*_ + *ν*_*π*_ = 3 + 3 from the fit to the Bodner et al., 2014 data) out of *n*_s_ = 128 total features (combining the 64 each of orthographic and phonological feature-spaces). As in Caplan and Guitard (n.d.), in our model of duration, we assume that after studying a pure-long list, participants can largely disregard the shallow (orthographic and phonological) features. Unlike production, increasing duration does not saturate and introduce cross-talk because the additional features are sparsely subsetted. Thus, after a nonmonotonicity at very small numbers of semantic features stored (left of the graph), the list-strength effect (*RoR*) increases swiftly above 1 and the mirror effect becomes increasingly pronounced. And unlike production, disregarding shallow features here helps the already-superior condition even more, but because disregarding only occurs on pure-long lists, this offsets some of the list-strength effect by further advantaging pure-long lists over long items in mixed lists.

### Graded Production

Forrin et al. (2012) found a rank-order of production: Vocalization > Whispering > Silence. Kelly et al. (2024) found that typing just two or three letters of a word resulted in mid-level recognition-memory between silence and typing the whole word. Our attentional subsetting account of production is compatible with these results. For *d*^′^ and both hits and false alarms (Figure 3a, c, red plots of mixed lists), more production leads to a monotonic improvement in all three performance measures as one moves from left to right (increasing *ν*_*π*,aloud_) over a range of attended features that is relatively small (but not sparse) compared to the production feature-space. When more than a few phonological features are stored per item, as already noted, the aloud condition levels off but the silent condition continues to be increasingly hurt by being present in a list mixed with aloud (or produced) items. When production is manipulated between lists, the production effect, itself, is more fragile, so a strict monotonic effect of graded production would not necessarily be predicted (blue plots). It is interesting to note that this is different for our model of duration, which predicts that more separation in duration will continue to separate performance of long and short items, both in mixed lists and in pure lists.

Our account of production may explain some of the finer details of the results of the two experiments reported by Kelly et al. (2024). In their Experiment 1, participants either typed all the letters of a word (one quarter of the words) or 3 letters of the word (half the words) or no letters (one quarter of the words. In Experiment 2, the middle condition demanded just 2 letters. Production (with these three levels) was manipulated in mixed lists. The hit rate was greater for the 3-letters-typed words in Experiment 1 than for the two letters-typed words in Experiment 2. However, in addition, the hit rate for the all-letters words and silent words were lower. The authors did not comment on these features (and they were not apparently produced by their simulated model). But this is what we expect; more production leads to greater effective strength of an item, but introduces more crosstalk via confusability in the production domain, increasing competition within a list. If the 3-letters items have more production-related features stored than the 2-letters items, they also compete more against the all-typed and nontyped items, reducing the hit rates of those two item-conditions. That said, this is an explanation of a between-experiment effect, which would need to be confirmed within a single experiment.

### Interaction Between Study Time and Production

Next we consider the effect of extending study time on the size of the production effect. In our formulation of stimulus duration, we assumed that earlier processed features are superficial, subsetted from a compact feature subspace, and later processed features are deeper, sparsely subsetted from a larger feature subspace. The implication is that the longer the participant studies an item, to a degree, the more sparsely subsetted, deep (e.g., semantic) features will be available to rely on. Immediately we shall see that the additional sparse features will increase performance but also will eventually outnumber the shallow features. It is the shallow features we assume are responsible for the production advantage. So for long durations, as performance increases, the relative advantage due to production will also reduce.

To visualize how all these effects interact, we simulated a simplified version of stimulus duration, with and without production. Although it is probably the case that phonological features are attended in the silent condition, for simplicity we fix *ν*_*π*,silent_ = 0 and *ν*_*π*,aloud_ = 3 as in our previous model of production that we fit to the Bodner et al. (2014) data. That is the entire implementation of production in this model version. Meanwhile, as delay increases, we assume both the number of processed orthographic features and the number of processed semantic features increases (linearly, for simplicity) and also for simplicity, we fix those rates to be identical. Thus, *ν*_*ω*_(*t*) = *ν*_*σ*_(*t*) = *rt*, where *t* is time in ms and *r* is the rate of processing of features per ms. The orthographic features will, however, be truncated at *ν*_*ω*_ = 8. They would have to be truncated at *n*_*ω*_ anyway, but it would seem more realistic if the orthographic features were never fully attended. The semantic features need no upper limit because they dwell within a much larger feature space and will never extend beyond the sparse regime anyway. As before, we assume that participants studying pure-silent lists largely disregard phonological features, and when tested on mixed lists, participants tune their response criterion to be the average of that used for aloud and silent items.

Figure 3g–i shows that if very few features are produced, a production effect is predicted for both pure and mixed lists, but with a substantial list-strength effect, a larger effect in mixed than in pure lists (*RoR* > 1). As more deep features are attended, performance increases and in fact takes drives increasingly more of correct performance. This eventually reduces the *RoR* to a value very close to 1; because semantic features are sparsely subsetted, they are rather immune from cross-talk due to other studied items (Caplan, 2023), so list composition exerts less effect on recognition. The corner in the plot is at the maximum value of *ν*_*ω*_ we set (8). Thereafter, only the attended semantic features, *ν*_*σ*_, increase in number. As they do so, the production effect persists and attenuates only very slightly. Panel i also shows that the nearly equivalent *d*^′^ values in pure lists are due to slightly different tradeoffs between hits and false alarms (compare the blue plots). The pure-aloud condition has fewer false alarms and more hits (a mirror effect) at nearly all simulated durations, similar to what Bodner et al. (2016) found.

In immediate serial recall, the effect of study time on the production effect reduces but does not eliminate the production advantage (Murray, 1965) but this remains to be tested in recognition. Although not direct, aligning with the prediction, Bodner et al. (2016) manipulated study time only of silent items and found that that narrowed the difference between produced and nonproduced items.

### Effect of Restricted Test Time

Kelly et al. (2022) manipulated the time available to participants to process and judge each test probe. They gave participants a long (5,000 ms) response deadline or a short (800 ms or 750 ms) deadline, manipulated within subjects but between lists (blocks). Production was manipulated within lists (mixed lists). The hit rate was reduced (and false-alarm rate increased) in the shorter deadline lists, but the advantage of aloud over silent items was comparable (∆hit rate = 0.20 and 0.17 for long and short deadline, respectively in their first experiment and 0.17 and 0.11 for Experiment 2). Without modification, our model makes the same prediction for test time as it does for study time, because masked-out features are set to zero. This multiplies through as zeroes whether the masked-out feature is part of the memory or part of the attended probe. Figure 3g–i thus could be reinterpreted as a prediction about the amount of test time—as long as the study time were at least as long as the test-phase response deadline. Consistent with the data, a sizeable effect of production on hit rate in mixed lists is produced at all study or test times. Our model does not explain the interaction, especially in their second experiment, where the advantage of production is greater at the longer deadline than the shorter deadline. Note that the persistence of the production advantage at short response deadlines is inconsistent with the assumption that production-related features are iteratively retrieved (Jamieson et al., 2016). Still, the wrong prediction about the interaction between production and response deadline shows us one way in which our very simple model of response deadlines is incomplete.

### Semantic Processing

The model thus far predicts an additional kind of finding. If participants were instructed to attend to deep or semantic features, for example by instructions to form visual imagery or with a deep level of processing as an orienting task (or even drawing; Fernandes et al., 2018), that may effectively shift the model rightward in Figure 3g–i. In other words, if participants explicitly attend deeper features, a production effect would still be present, but could be attenuated compared to a control condition.

Suggestive of this, MacLeod et al. (2010) found a robust production effect when participants were given animacy judgements as an orienting task (their eighth experiment), but without a direct comparison to a different orienting task or no orienting task, we cannot know if the production advantage was relatively attenuated. Taikh and Bodner (2016) came closer. In a between-subjects manipulation of production, participants had to imagine what the word meant or imagine the word, itself, in capital letters. The imagery manipulation was done within lists. Compared to their other experiments that manipulated font size and generation, the production effect was far smaller when participants used imagery-based strategies (their third and fourth experiments).

### Proceduralism Through the Lens of Feature Spaces

The *Distinctiveness Heuristic* was proposed by MacLeod et al. (2010), following Dodson and Schacter (2001), to function not at the feature level but via conscious recollection. The idea is that the act of production, itself, is stored in memory. Then, the participant can use a heuristic whereby, if that act of production comes to mind, associated with the probe item, that can be used as evidence that the word was studied. MacLeod and colleagues, starting with MacLeod et al. (2010), have noted that this captures the spirit of Kolers’ proceduralist theory (Kolers et al., 1973). The idea that participants draw upon memory of the actions used while studying a stimulus was in Kolers’ theory but Kolers thought of this not all-or-none, but proposed that participants encoded specific action-related features. This was proposed for the production effect by Forrin et al. (2012). Subsequently, Jamieson et al. (2016) implemented this kind of adaptation of the distinctiveness heuristic mathematically, in a somewhat less explicit way. Their assumption was that evidence of action/production dwells within the item representation, but needs to be recovered through an iterative retrieval process. However, a problem for this account is that the lengthened response times anticipated by this mechanism are not observed, and a fast response deadline does not come close to eliminating the production advantage (Kelly et al., 2022). MacLeod et al. (2010) also differed from proceduralist theory, along with Dodson and Schacter (2001), by proposing the heuristic is applied consciously. but Kolers repeatedly asserted his view that recovery and comparison of action features was not conscious.

We retain the concept of distinctiveness and production-related action features, but stick closer to Kolers’ feature-level view and not assuming conscious application of a rule is required. We suggest that the act of production draws attention to (and encoding of) features related to actions involved in production. Thus, in addition to the *n*_*π*_ phonological features, we assume there is a separate subset of *n_α_* features (where *α* stands for “action” features). For typing, the production space will undoubtedly be highly driven by the spatial positions of the keys on the keyboard, hand and finger used, etc. (MacNeilage, 1964). For the more classic, vocal production, the features consist of the movements and sensations of the mouth and vocal system. The similarity structure of the *α* subspace will have some commonality with the similarity of the *π* subspace (or *ω* subspace, in the case of typing) but it will not be identical (just as for the relationship between the orthographic and phonological subspaces). In this way, production can be seen to add some feature-level distinctiveness and similarity that is redundant with the stimulus-feature space (phonological, in this example) but it will also add some distinctiveness and similarity contributed by the action-space features that is not echoed in the stimulus features.

One implication of this implementation of proceduralism is that it still could lead to sizeable production effects in pure lists or between subjects, contradicting the intuition Taikh and Bodner (2016) had with respect to the *Distinctiveness Heuristic*. It also implies that the advantage due to production should decrease with increasing list length, as the production feature space becomes more fully occupied, and as duration increases and semantic or other deeper processing takes over.

### Homophone Lures

Next we address a recent finding that would seem to challenge the core idea we inherited from other models of the production effect. Fawcett et al. (2022) conducted a very clever experiment aimed at testing the idea that production only influences encoding of features that are directly related to production. Participants made two-alternative forced choice judgements (2AFC) between a target and a lure based on mixed-production lists. One group of participants always had randomly selected words as lures (“standard lures”). The other group received only homophones as lures (such as towed vs. toad). Because homophones cause participants to make the identical sounds (phonological features) as each other, they reasoned that if vocal production only influences the encoding of phonological features, there should be zero production advantage for homophone comparisons. Because those additional phonological features would be identical for the homophone lure and the target, they should offer no net advantage for production. Contradicting this, a large production effect was observed for participants in the homophone-lure condition. This effect was close to the same magnitude as for participants in the standard lure condition. The authors viewed these findings as challenging the idea that production only influences the encoding of production-related (superficial) features. They argued that production must therefore not (or not only) increase encoding of production-related features like phonological information, but also enhance semantic encoding of produced items.

**Figure 5:**
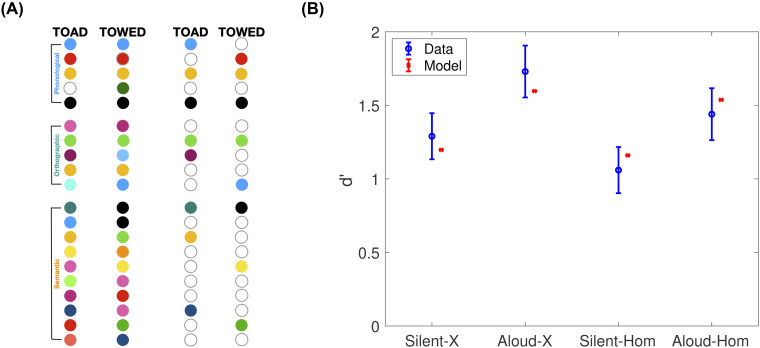
(A) Schematic illustration of how item-specific attentional subsetting can disambiguate homophones. Depicted are hypothetical vector representations of TOAD and TOWED (left) and attentionally subsetted versions of those vectors (right). We assume a pair of homophones have identical values of all the phonological features as each other (phonological features, top). We also assume that the spelling will be similar, so many orthographic features will also match (middle feature space). The semantic features will tend to be quite different. Because attentional subsetting is item-specific, by chance some of the same phonological features will be attended on the two words, resulting in encoded features that are not diagnostic on a forced choice between the two. But other features will be attended for one but not for the other, and the pattern of zero-valued features does afford some diagnosticity. (B) Best-fitting model plotted alongside the data from the homophone study reported by Fawcett et al. (2022). Silent-X and Aloud-X are the data for participants who judged targets against standard lures. Silent-Hom and Aloud-Hom are the data for participants who judged targets against homophones.

A central assumption of attentional subsetting theory may provide a third account of the homophone experiment that still does assume that production increases the number of production-related feature encoded (such as phonological features), but does not require any difference in encoding of semantic features. That is, we assume that not all phonological features are stored, and the subset of features stored (in any feature subspace, but including phonological features) is item-specific. The idea here is that despite the participant producing the same phonemes, they will store a different subset of those phonemes while pronouncing toad than towed (illustrated in Figure 5a). In other words, the orthographic features and semantic features that differentiate towed from toad will also draw attention to different subsets of the phonemes. Even if there is considerable overlap between those subsets, and despite the fact that those overlapping features will also have the exact same values, the nonoverlapping portions of the phonological-space masks may be sufficient to add diagnosticity to the recognition judgement. In sum, the additional phonological features, where they overlap, will increase the amount of similarity-based cross-talk across list items, reducing accuracy, but the addition of the nonoverlapping, item-specific features will offset this reduction by improving the discriminability of the two homophones.

One additional observation may help to explain the homophone findings. From visual inspection of the homophone stimuli used by Fawcett et al. (2022), it is clear that they possess quite a lot of orthographic similarity, albeit less than phonological (the example, towed and toad, have three identical letters, also in the same relative order). The reason silent items are duped by homophone lures, therefore, could be because of that orthographic similarity, or it could be due to subvocalization, the participant imagining saying the word. Subvocalization is quite plausible given that the lists were mixed and there was no experimentally induced articulatory suppression.

We implemented the homophone paradigm in a simulation adapted from the one we used to fit the Bodner et al. (2014) data. The feature space sizes were retained. *L* = 80 words per list. All phonological features were assumed to be identical for the homophone probes. Again, *ν*_*π*,silent_ = 0. We searched four free parameters. *ν*_*π*,aloud_ was varied from 1 to 16. *ν*_*σ*_ was varied from 1 to 16. The number of identical orthographic parameters for homophones was varied from 1 to 32.^4^ Finally, *ν*_*ω*_ was varied from 1 to 8. The best-fitting model, plotted in Figure 5b, had a log-likelihood = 17.88, *ν*_*σ*_ = 4, *ν*_*ω*_ = 7, #identical orthographic features for homophones = 32 and *ν*_*π*,aloud_ = 4. These are close, but slightly greater in terms of numbers of features fit to the Bodner et al. (2014) data. Especially the greater number of semantic features is consistent with the longer study time per word here (4 s compared to 2.5 s). Even with four free parameters, the fit is not perfect. However, the surprising result, a large (comparable-sized) production effect for the homophone group, was easily captured by the model. The main qualitative feature missed by the model is that it underestimates the lower *d*^′^ for the homophone group. However, that effect is present, although quite small. Although this is a quantitative deficiency, it is unclear if this is a major miss by the model, because the comparison, standard versus homophone, is between-subjects, and the two groups of participants may have approached the task differently. Still, the model fit stands as proof of principle that a large production advantage can be expected for target–homophone comparisons even if one assumes that production only affects encoding of phonological features and does not have any effect on semantic features. To be fair, we have implied not that semantic processing is irrelevant, but that it is the same for both aloud and silent words, and semantic processing modulates encoding of phonological features.

## Discussion

We have built on other mathematical models of production that assume that production results in more phonological (or orthographic, in the case of typing or writing) features being stored in memory (Jamieson et al., 2016; Kelly et al., 2022; Saint-Aubin et al., 2021). We extended this view by integrating additional assumptions from attentional subsetting theory (Caplan, 2023; Caplan & Guitard, n.d.; Caplan et al., 2022), that only a small subset of features are attended and thus encoded within any given feature subspace, that those subsets are item-specific and often will reiterate quite well, especially at test, and that phonological and orthographic features are attended earlier than semantic features and are excerpted from low-dimensional subspaces as opposed to high-dimensional semantic and imagery subspaces.

This reconciles the large list-strength effects in recognition, that are found with production, with the very small or null list-strength effects found with other manipulations of *strength*, especially stimulus duration and spaced repetitions. Other findings that are compatible with the theory included graded effects of amount of production on the production effect, the effects of both study time/presentation rate (suggestive in Bodner et al., 2016; Murray, 1965) and test time/response deadline (Kelly et al., 2022), the effect of deep encoding instructions (MacLeod et al., 2010; Taikh & Bodner, 2016) and the finding of a large production effect even for homophone lures (Fawcett et al., 2022).

Our theory of production shares some characteristics with each of the two major propositionally formulated theories: Strength Theory and the Distinctiveness Heuristic. Next we relate our theory with each of those in turn, and then discuss broader implications and predictions that follow from the theory.

### Strength Theory

In their landmark paper, MacLeod et al. (2010) reference the so-called *null list-strength effect* in recognition as a major argument against Strength Theory of production:

We are convinced of their difference because of a striking dissociation: The list-strength effect does not occur in recognition, yet the production effect has been observed primarily in recognition, where it is large and easily obtained. This is important because the fact that relative strength does not affect recognition despite the production effect being solid in recognition indirectly suggests that the production effect is not due to relative strength. (p. 681)

From our perspective, this is rather backwards. Caplan (2023) noted that list-strength effects that were described as null were generally slightly positive (*RoR* > 1, often around 1.1), albeit nonsignificant. So the first point is that those findings are more accurately described as near-null list-strength effects. The challenge to theory is really to explain why the list-strength effect is so small, not why it is strictly absent.

With the attentional subsetting framework, Caplan (2023) reformulated list-strength effects as continuum phenomena. The small magnitude of those effects was seen as resulting from the stronger condition (e.g., repeated presentation of items) adding features that were subsetted from a high-dimensional space, leading to sparse functional representations encoded in memory, which introduced very little additional cross-talk interference compared to the weaker condition (see our model of duration here; Figure 3, top and bottom panels). This explanation also presumed that there is cross-talk due to shallower features such as orthographic or phonological features, but that those features were comparably present in the strong and the weak conditions; “strength” manipulations do not increase or decrease that source of interference from other list items.

In this account, the production effect is different because it improves memory by encoding more superficial features, that cannot be sparsely subsetted and thereby introduce more cross-talk interference in the better (produced) condition. In other words, the production effect functions the way that previous researchers such as Ratcliff et al. (1990) thought strength should function. So MacLeod et al. (2010) should have argued not that production is not strength, but that previous strength manipulations were not functioning like strength manipulations. Rather, production does function as one would expect of a manipulation of strength, namely, producing a substantial positive list-strength effect. Later, Jamieson et al. (2016) were duly confused about the strength logic, writing

Received wisdom is that the distinctiveness account predicts a much stronger mixed-list than pure-list production effect whereas a strength account predicts equally probable and equally sized mixed-list and a pure-list production effects (see MacLeod et al., 2010, p. 160).

They then go on to implement an “OG” strength model and naturally, it produces a list-strength effect (see also the demonstration by Caplan, 2023). They conclude, in fact, that both Strength Theory and distinctiveness lead one to predict a list-strength effect with production—which matches the data. Therefore, they warn that this demonstrates that strength and distinctiveness are challenging to select between experimentally. They missed the way in which the null list-strength effect story had bent over backwards, but from a different angle than MacLeod et al. (2010). Jamieson et al. (2016) came closer to our view, that production manipulates strength and with the implication that classic “strength” manipulations were weird. But in fact, their implementation of production, additional encoded production-related features, captures both strength (through functional vector-length) and distinctiveness (the additional functional dimensionality afforded by those longer functional vectors) with one mechanism. We viewed this as an elegant theoretical proposal, and adopted it. Although we added assumptions, this concept remains at the center of our account of the production effect.

Finally, the framing of near-null list-strength effects as strict-null effects, starting with Ratcliff et al. (1990), led modelers to develop models that would automatically produce null list-strength effects. The most influential of these is REM (Shiffrin & Steyvers, 1997). REM assumes that each memory is stored in a separate, “local” memory trace. A recognition probe is matched to each local trace, computing a likelihood ratio based on both the matching and mismatching features, and then these are averaged across all traces to produce the evidence used to decide on the response (old vs. new). They also assumed that strengthening results in more (correct) features stored in the item’s trace. This produces a differentiation effect, where a stronger trace both matches itself more when presented as a target, and matches other (lure) items less because of the presence of additional features that could mismatch those lure stimuli. Among other things, this produces the effect they desired: negligible influence of the strength of one item on recognition of another, hence a null list-strength effect. However, in accommodating null list-strength effects, one must be careful that the model has not to lost the ability to predict substantial list-strength effects when they are observed. Interestingly, Kelly et al. (2024) fit their data with REM, and although they were rightly pleased that it could explain increased hit rates for items that were produced more (a graded effect, discussed earlier), their simulated model produced nearly no within-list competition effects. In their data, when the middle condition was produced more (3 letters typed rather than 2), hit rates of both the all-typed and nontyped reduced, differing from their REM-based model. As noted by Caplan (2023) and Caplan and Guitard (n.d.), a continuum account, such as ours, has the chance to explain a diverse range of magnitudes, and even directions, of list-strength effects. REM does have ways of producing list-strength effects, such as by storing multiple traces of an item rather than strengthening a single trace (Ensor et al., 2021; Shiffrin & Steyvers, 1997). But another approach might be to incorporate our assumptions about feature subspace characteristics into the representations used in REM, which might produce large production list-strength effects due to cross-talk because production-features increase similarity across traces.

### The Distinctiveness Heuristic

As authors like Jamieson et al. (2016) have already noted, storing more features does generally increase distinctiveness. This speaks to the general concept of distinctiveness. But the heuristic referred to something quite different: a process more akin to *recollection* in dual-process theories of recognition (Yonelinas, 1999). MacLeod et al. (2010) drew a connection to Kolers’ proceduralism, but Kolers explicitly assumed proceduralist effects were not deliberate or conscious. Both recollection/re-experiencing and proceduralism have an air of mystique around them. But attentional subsetting theory provides a very nonmystical, uncomplicated and concrete way these proceduralist-like effects might come about. Namely, production draws attention to features that dwell within a production action feature space (or possibly a combination of action-features, phonological, orthographic and mappings amongst them, which could be adjudicated in future experiments). That feature space usually bears some similarity to its corresponding stimulus-feature space, but they are not strictly equivalent. For this reason, production-space features may afford additional distinctiveness beyond the distinctiveness present in the stimulus feature-space. This idea could be tested in future experiments. For example, similarity-based errors due to keyboard position of letters (MacNeilage, 1964) should be more frequent in typed than nontyped conditions. The prediction is not so clear-cut, though, because at the same time, typed words achieve higher accuracy.

Jamieson et al. (2016) had an equally nonmystical implementation of proceduralism, where production-related features were retrieved iteratively while processing a probe. This leads one to expect longer response times for pure-aloud lists than for pure-silent lists, but such effects are not found and fast response-deadlines do not seem to eliminate the production advantage (Kelly et al., 2022). Interestingly, when we manipulated stimulus duration, participants often produced longer response times for the longer condition (Caplan & Guitard, n.d.). This was consistent with our assumption that longer study time leads participants to process more deep features that are sparsely subsetted. Thus, taking more time at test will pay off in terms of a speed-accuracy tradeoff; taking longer to process those additional deeper features will be likely to support better recognition accuracy. When items are studied for less time, processing the probe longer has diminishing returns. From the same perspective, if production enhances memory accuracy by encoding additional phonological or orthographic features, those are features that are processed early, so there is little reason to predict longer response times to recognize produced items.

### Limitations and Future Directions

#### Tradeoffs Across Feature Spaces

Saint-Aubin et al. (2021) explained production effects in serial recall partly as a modality effect, supplementing visual processing with auditory processing of words. But the second major element of their model account was the assumption that production displaces rehearsal, itself known to improve memory. Conceivably, production may displace other processes, as well, that would otherwise have benefitted memory, such as deep levels of processing like visual imagery and semantic elaboration. This notion could be incorporated into the attentional subsetting framework as applied to recognition memory. It could lead to more nuanced predictions. Offsetting some of the benefits to recognition memory of production due to increasing the number of encoded stimulus-features, production may reduce the number of orthographic or even semantic features attended, as illustrated in Figure 1. The prediction might be similar to those we presented here, but the production advantage may be further reduced as semantic or imagery processing, or even multi-item rehearsal or associative processing, become more feasible for participants, such as when study time increases.

#### Compatibility With Other Models

Our continuum view is quite flexible and anticipates (as well as postdicting) a few dependencies of the production effect on other variables such as study time, list length, and study strategy. Although we implemented the ideas in a distributed, global-matching model, the same principles could be implemented in any model with a vector representation of items, including local-trace models (note that MINERVA 2 and the Feature Model, which have been applied to the production effect, do not normally produce null list-strength effects, different than REM). That said, the distributed model also has a lot of flexibility. With the sparse-subsetting assumption, it can produce several of the phenomena that local traces were invoked in part to solve, such as the apparent null list-strength effect and (combined with differentiation), pronounced strength-based mirror effects. Sparseness achieves what the local traces, along with differentiation, achieve, that matching is carried out with little cross-talk across studied items.

### Conclusion

We view production effects not as a set of phenomena that need customized theoretical accounts, but as a special case of attentional subsetting theory, where the pecularities of production-related memory are assumed to be driven by the peculiarities of the feature space that production acts upon. This led us to reconsider production as a form of memory strengthening. As a particular case of “strength,” our account unifies production with other strength manipulations like stimulus duration and repetition, explaining how they differ. As such, our theory both addresses the production effect in recognition and provides a framework for understanding a broad range of experimental manipulations through the lens of attended feature subsets and their respective dimensionality. Our specific application of the theory to the production effect in recognition memory assumes little more than that production results in additional, production-related features stored in memory, borrowing from other models of production effects (Jamieson et al., 2016; Kelly et al., 2022; Saint-Aubin et al., 2021). The assumptions of attentional subsetting theory add to this: (1) that small subsets of features are encoded, (2) that the subset attended during test will tend to be similar to the subset attended during the study phase, (3) that production increases encoding of shallow, nonsparsely subsetted features, whereas otherwise, additional study time increases encoding of deeper, sparsely subsetted features. Numerous empirically observed phenomena are produced with just these assumptions, even when implemented in an overly simplified vector-summation model, but could easily by incorporated into virtually any model that assumes some kind of vector representation of items. This account is quite different from the dominant propositional theories of production effects, Strength Theory and the Distinctiveness Heuristic, but embodies some desirable attributes of each. This simple view of production avoids the need for more complex or strategic accounts, including suggestions that production enhances encoding of features beyond superficial features related to production. Finally, by providing some contrasting empirical phenomena, the production effect has extended the generality of attentional subsetting as a theoretical framework for understanding memory.

## Footnotes

^1^
We do not make strong claims about whether such attention is automatic or conscious, but rather, stick to the mathematical formulation of the model.

^2^
This variance ensures that on average, the vector length will be 1, so vectors are approximately (but not strictly) normalized, especially when *n* is very large.

^3^
We will use the words “deep” and “semantic” interchangeably, but there is more nuance. “Deep” refers to features that take longer to access than “shallow” features, and from a larger feature space. Deep features could include features related to meaning, but also related to things like imagery or even associations between studied items. Conversely, some semantic features might be accessed as quickly as orthographic and phonological features, and perhaps from a small, compact semantic subspace. We use these terms simplistically to keep the exposition clear but this nuance will need to be tackled in the future.

^4^
It fit at 32. We considered raising the search range but felt that too much orthographic similarity would deviate from the spirit of this account so we left the cap at 32, one half of the total number of orthographic feature space.

## Notes

**Open Science:** MATLAB code used to generate plots of the analytic derivations is available at https://osf.io/7x52q/ (Caplan & Guitard, 2024).
